# Obstetrics and gynecology clerkship for males and females: similar curriculum, different outcomes?

**DOI:** 10.3402/meo.v18i0.21506

**Published:** 2013-12-10

**Authors:** LaTasha B. Craig, Chad Smith, Sheila M. Crow, Whitney Driver, Michelle Wallace, Britta M. Thompson

**Affiliations:** 1 Section of Reproductive Endocrinology and Infertility, Department of Obstetrics and Gynecology, University of Oklahoma Health Sciences Center, Oklahoma City, OK, USA; 2 Department of Obstetrics and Gynecology, University of Oklahoma Health Sciences Center, Oklahoma City, OK, USA; 3 Clinical Skills Education and Testing Center, University of Oklahoma College of Medicine, Oklahoma City, OK, USA; 4 Office of Medical Education, University of Oklahoma College of Medicine, Oklahoma City, OK, USA

**Keywords:** clerkship, gender, grades, obstetrics and gynecology clerkship, performance

## Abstract

**Objective:**

To determine if performance differences exist between male and female students on a 6-week obstetrics and gynecology (Ob/Gyn) clerkship and to evaluate potential variables that might underlie any observed variations.

**Study Design:**

Final clerkship grades and component scores (clinical evaluations, objective structured clinical examination [OSCE], oral examination, and National Board of Medical Examiners [NBME] subject examination) from July 2007 to June 2010 were matched by student and analyzed by gender. Basic science grade point average (GPA) and initial United States Medical Licensing Exam (USMLE) Step 1 scores were used to establish students’ baseline medical knowledge. On a post-clerkship questionnaire, a subset of students reported the numbers of procedures they performed during the clerkship; students also completed online pre- and post-clerkship questionnaires reflecting their self-assessed confidence in women's health clinical skills.

**Results:**

Scores were analyzed for 136 women and 220 men. Final clerkship grades were significantly higher for females than for males (89.05 vs. 87.34, *p*=0.0004, *η* ^2^=0.08). Specifically, females outscored males on the OSCE, oral, and NBME subject examination portions of the clerkship but not clinical evaluations. Males reported completing fewer breast examinations (*p*=0.001, *η* ^2^=0.14). Pre-clerkship, males were significantly less confident than females in women's health clinical skills (*p*<0.01) but reached similar levels upon completion of the clerkship. No gender differences were detected for basic science GPA and USMLE Step 1 scores.

**Conclusion:**

Student gender is associated with final grades on an Ob/Gyn clerkship. Further research regarding these differences should be explored.

Research has shown that female students tend to outperform their male counterparts in the obstetrics and gynecology (Ob/Gyn) clerkship with respect to final grades (1–3). However, the effects of gender on individual assessment components are much less clearly delineated. Moreover, few studies have evaluated factors associated with these differences.

More than a decade ago, Bienstock and associates found that female students performed better overall and, specifically, on the written examination and the objective structured clinical examination (OSCE); however, clinical evaluations showed no significant differences (4). More recently, one study observed no gender difference in United States Medical Licensing Exam (USMLE) scores, but noted that female students perform better in Ob/Gyn and Internal Medicine clerkships (5). Two other studies – conducted at a single school – suggest once MCAT and GPA variables are controlled, females still outperform males on every component of a clinical skills examination, and score significantly higher on history-taking and physical examination skills (6, 7).

Since initial studies (4), there have been substantial changes in Ob/Gyn clerkships – including increasing numbers of female residents and faculty (resulting in more female evaluators) (8), more extensive use of simulation such as OSCEs (9), and better defined clerkship competencies at the national level (10). Thus, it is both timely and important to reexamine these findings and expand our focus to potential mitigating factors underlying any observed differences.

Reasons for significant and persistent gender differences remain veiled. In two studies, males reported that their gender negatively affected their experience during the Ob/Gyn clerkship (11, 12) – one of which (12) found a concurrent positive gender effect for females. This same study (12) suggested that males felt their gender limited their abilities to excel on the clerkship and that more procedural experience went to their female classmates (12). Despite this perception, differences in numbers of procedures have not been demonstrated.

The purpose of our study was to determine if females, even a decade later, continue to outperform males on the Ob/Gyn clerkship and, if so, to further examine various measures that might be correlated. Based on previous studies, we hypothesized that females would perform similarly to males in the first 2 years of medical school but would perform more procedures than males during the clerkship, and have higher clinical self-confidence in the area of women's health than males.

## Materials and methods

To determine if differences exist in course performance between male and female students on a 6-week Ob/Gyn clerkship at the University of Oklahoma College of Medicine, retrospective data were analyzed for 356 third-year medical students completing the Ob/Gyn clerkship in Oklahoma City between July 2007 and June 2010. Data included the overall clerkship numeric grades, clinical performance evaluations by residents and faculty, oral examination scores, OSCE scores, and National Board of Medical Examiners (NBME) Ob/Gyn subject examination scores. To establish students’ baseline medical knowledge, we retrospectively evaluated differences in basic science GPA (overall numeric grades from first and second year of medical school) and initial USMLE Step 1 scores, clerkship rotation sequence, and academic year. All data were matched within students prior to analysis.

To determine gender differences in the number of procedures performed, students completing the clerkship between August 2009 and June 2010 (*n*=105) were asked to complete an online post-clerkship questionnaire regarding the number of procedures they performed (procedural experience). On a brief questionnaire, students (via self-report) indicated the numbers of patients interviewed, speculum examinations performed, breast examinations performed, and vaginal deliveries in which they participated during the clerkship. They were asked to select from the following options: 0 (none), 1 (1–2), 2 (3–5), 3 (6–8), 4 (9–11), 5 (≥12). To evaluate if female students were more confident in their women's health clinical skills, the same students were asked to complete an online questionnaire before and after the clerkship assessing their confidence in this area using a 1–9 scale (1 = poor, 9 = excellent) with six items. Pre- and post-test data were appropriately matched, and mean confidence was determined for all six items.

Institutional Review Board approval was obtained from the University of Oklahoma Health Sciences Center. Data analysis included both descriptive and inferential statistics – using parametric and non-parametric tests, as appropriate. Effect size was gauged using eta squared, which reflects magnitude via the percent of variance accounted for. We used recommended guidelines for determining small (0.01), medium (0.06), and large (0.16) effect sizes (13). The Pearson Product Moment correlation coefficient (*r*), or the non-parametric equivalent, assessed the strength of relationships between variables. *α* was set at 0.05. All data were analyzed using SPSS 19.0.

## Results

For this study, we retrospectively analyzed all students completing the Ob/Gyn clerkship from July 2007 to June 2010 for a total of 356 records (females = 136, males = 220). All records were complete, and therefore, none were excluded. We noted no significant variability in the clerkship data by rotation sequence or academic year. As shown in Table 1, final overall clerkship grades for females were higher than those for males (89.05 vs. 87.34, *p*=0.004). Effect size analysis indicated that these differences were of medium educational significance (*η* ^2^=0.08). When we assessed differences in each component of the overall grade, we noted that females outscored males on the OSCE, oral examination, and the NBME shelf examination but not on clinical evaluations (Table 1).

**Table 1:** Obstetrics and gynecology clerkship performance between males and females (females = 136, males = 220)

Student performance	Females (95% CI)^a^	Males (95% CI)	*p* b
Clinical evaluations	91.7 (90.9–92.6)	91.3 (90.7–91.9)	0.41
NBME^c^ shelf examination	75.3 (74.0–76.5)	73.0 (71.9–74.1)	0.01
Oral examination	91.5 (90.6–92.4)	90.2 (89.3–91.0)	0.04
OSCE^d^	88.1 (86.8–89.4)	85.8 (84.6–87.1)	0.02
Overall clerkship grade	89.1 (88.2–89.9)	87.3 (86.6–88.1)	<0.01

a 95% CI = 95% confidence interval.

b Multivariate analysis of variance.

c NBME = National Board of Medical Examiners.

d OSCE = objective structured clinical examination.

To ensure that these gender differences were not attributed to baseline knowledge differences, basic science grade point average (GPA) from the first 2 years of medical school and USMLE Step 1 scores were analyzed (*n*=356). Data revealed that males and females had comparable basic science GPAs from the first 2 years of medical school as well as USMLE Step 1 scores (*p*>0.05).

**Fig. 1:**
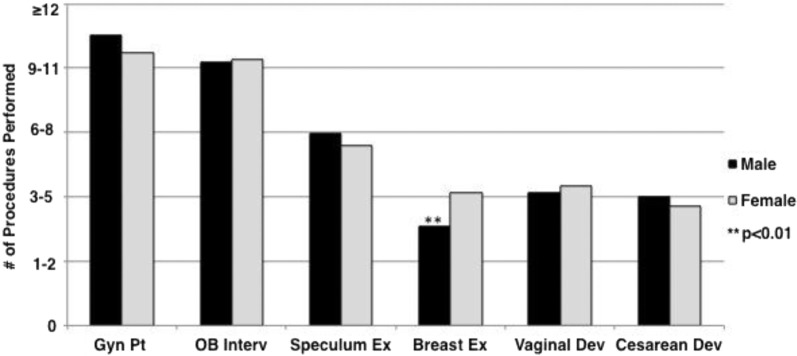
Procedures performed: medical students self-reported number of procedures completed during an Ob/Gyn clerkship (males = 42, females = 31). Gyn, gynecology; OB, obstetric; Interv, interview; Ex, examination; Dev, delivery.

We hypothesized that additional opportunities for female students to practice and participate in Ob/Gyn procedures may have contributed to these gender differences. Toward this end, students participating in the clerkship from September 2009 to June 2010 were asked to fill out a questionnaire upon completion of the clerkship. Response rate was 69.5% with 73 of the 105 students responding (43 males and 30 females). Analysis indicated an overall difference in the number of procedures completed between male and female students (*p*=0.033, *η* ^2^=0.19). However, post-hoc analyses found only one statistically significant difference: females reported performing more breast examinations than did males (*p*=0.001, *η* ^2^=0.14) (see Fig. 1).

**Fig. 2:**
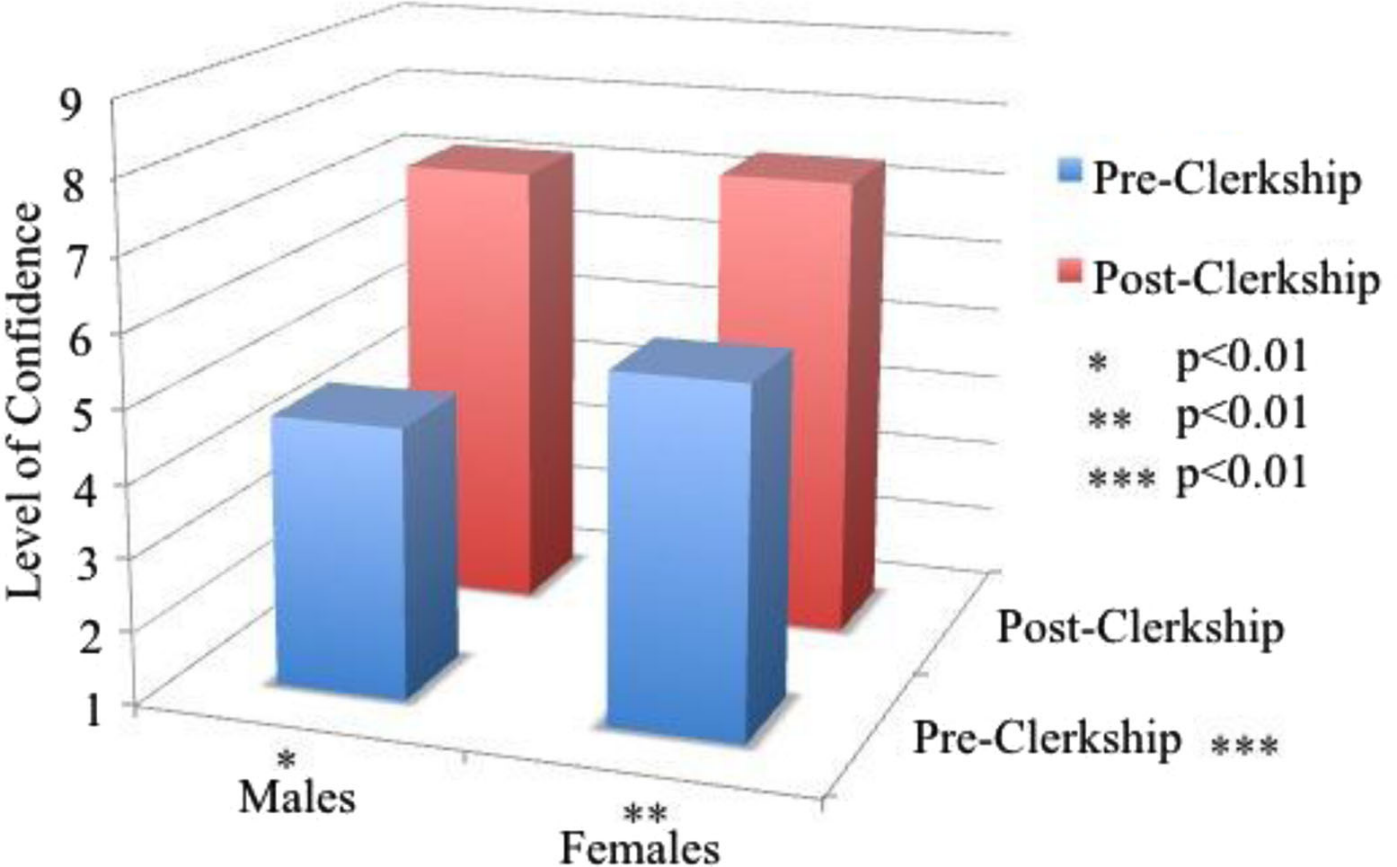
Self-confidence in women's health: ratings of confidence in women's health skills pre and post-clerkship by male (*n*=51) and female (*n*=37) medical students (1–9 scale; 1 = poor, 9 = excellent).

Finally, we tested the hypothesis that female students have more self-confidence in the area of women's health skills. Eighty-four of 105 students (response rate = 80.0%) completed both pre- and post-clerkship questionnaires (49 males and 35 females) regarding self-confidence in women's health clinical skills. Post-test analysis indicated no differences in self-confidence in women's health between the two genders. For both genders, confidence increased from the beginning to the end of the clerkship (*p*<0.01, *η* ^2^=0.67). Interestingly, post-hoc analysis indicated that males gained the most confidence compared to their female counterparts (*p*=0.005, *η* ^2^=0.09), reaching similar levels upon completion of the clerkship (see Fig. 2).

## Discussion/conclusion

Reexamining the relationship between gender and performance on the Ob/Gyn clerkship, our study found continued support of the hypothesis that females outperform males on the Ob/Gyn clerkship – similar to the findings of Bienstock and colleagues more than 15 years ago (4). Our results further showed that females outperformed their male counterparts on the OSCE, oral examination, and the NBME shelf exam. However, clinical evaluations were similar between males and females – despite having the increasing prevalence of female evaluators (8). In this study, 17 of the 23–24 residents were female – as was 50–60% of the faculty.

Once we confirmed the gender discrepancy in clerkship performance, we set out to identify confounding factors. We did not identify a gender discrepancy in basic science grades in the first 2 years of medical school or in USMLE Step 1 scores, suggesting that clerkship performance differences were not attributable to baseline knowledge between males and females.

The literature documents a strong perception among medical students that gender influences experiences on the Ob/Gyn clerkship (14). Chang and associates found that male students were more likely to report feeling that their gender negatively impacted their clerkship experience (11), and that males reported more patients refusing to allow them to participate in the physical examination. However, there was no significant gender difference in the reported number of examinations performed (11) – a finding confirmed by Akkad and colleagues (15). In contrast, a study by Powell and associates found that male students, compared to their female classmates, performed fewer breast and pelvic examinations during their third and fourth years of medical school (16). Our study only showed that females reported performing more breast examinations than did male students.

Although studies linking higher self-efficacy to better academic performance (17, 18) have shown that females, on average, are typically less self-confident in their clinical skills than are males, few studies have assessed confidence in skills related to women's health issues (19). As anticipated, our data indicated females were more confident in their abilities entering the clerkship. However, over the clerkship, both male and female students gained confidence in women's health-related skills and, ultimately, achieved equal levels of confidence in this area – making this an unlikely contributor to clerkship performance.

Our study findings are limited by several factors. First, the subjects represent data from a single institution – though our findings do concur with those from previous studies. Second, a more ‘objective’ measure of procedural experience might be preferable, since the accuracy of students’ self-reported procedural experiences is unknown. However, our curricular logging system requires that only the minimum number of required procedures be entered; as a result, students typically do not log procedures beyond the specified threshold.

In conclusion, we found that female students outperformed male students in the Ob/Gyn clerkship, specifically on the OSCE, oral examination, and NBME subject examination. Our results further suggest that these differences were not due to baseline performance during the first 2 years of medical school or to the number of procedures performed while on the clerkship. Both genders gained significant confidence in women's health-related knowledge and skills during the clerkship and achieved similar confidence levels upon completion of the clerkship, suggesting that this, too, did not contribute to the observed differences.

Future studies should further explore the reasons underlying these gender differences in performance on the Ob/Gyn clerkship that we, and others, have documented. In particular, it may be useful to investigate the potential of gender bias in assessment (i.e., gender of the evaluator), the quality and/or quantity of clerkship teaching experiences, or perhaps the level of effort or involvement of students during the clerkship. Future research might also draw comparisons with other clerkships, specifically, those that have historically been male-dominated (e.g., surgery) or those that may be similarly ‘female friendly’ (e.g., pediatrics).

Admittedly, our study has shown the absolute difference in scores between males and females to be small but persistent – confirming the results of previous studies. However, while only 5–6% of medical graduates ultimately enter residencies in Ob/Gyn, the vast majority will go on to provide care to female patients, regardless of specialty area; therefore, it is important that all medical students (both males and females) gain adequate clinical competence in women's health. In a field that is seemingly less attractive to male graduates (20), identifying the root of gender differences in Ob/Gyn training may help elucidate and, perhaps, even reverse this trend.
